# Supplementary material for: High-Fat Diet Impairs Mouse Median Eminence: A Study by Transmission and Scanning Electron Microscopy Coupled with Raman Spectroscopy
Source: Int J Mol Sci. 2021 Jul 28;22(15):8049. doi: 10.3390/ijms22158049 (PMC8347199; doi:10.3390/ijms22158049)
Supplement: Supplementary file 1 [file ijms-22-08049-s001.zip › Supplemetary Figs and Captions.pdf]

Supplementary Figure 1

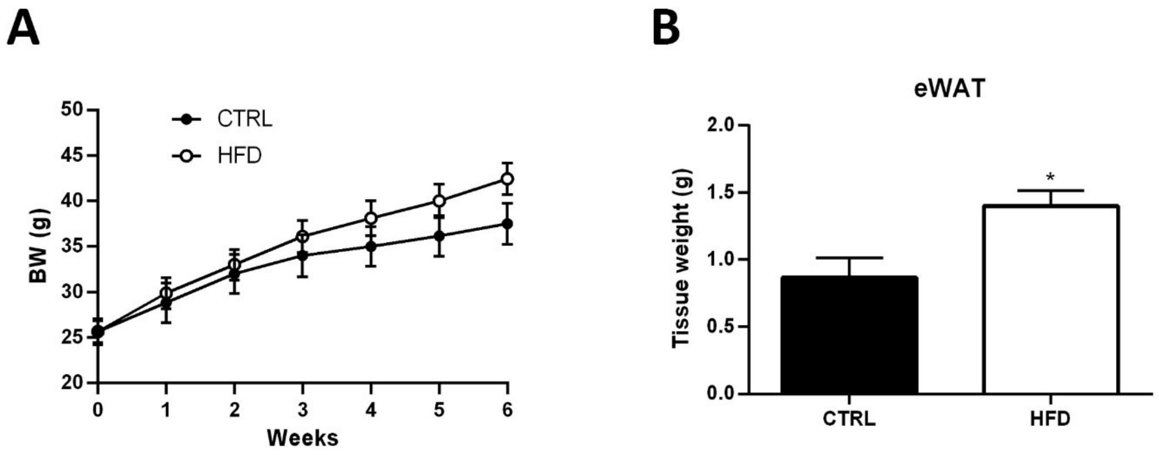

C

Casuistry Database: Arc-ME complex from 3 CTRL and 4 HFD mice.

Case	Administered Diet	No. maps	No. spectra
1	CTRL	1	616
2	CTRL	1	2139
3	CTRL	1	854
4	HFD	1	2378
5	HFD	2	938
6	HFD	1	630
7	HFD	1	756

Total number of maps, 8; total number of spectra, 8311.

Supplementary Fig. 1: A and **B:** body weight (BW) and epidydimal adipose tissue (eWAT) weight in CTRL and HFD mice. Data (n=6 CTRL, n=9 HFD) are mean ± standard error, *$p < 0.05$ (unpaired student's t-test). **C**: casuistry database for Raman spectroscopy analysis.

Supplementary Figure 2

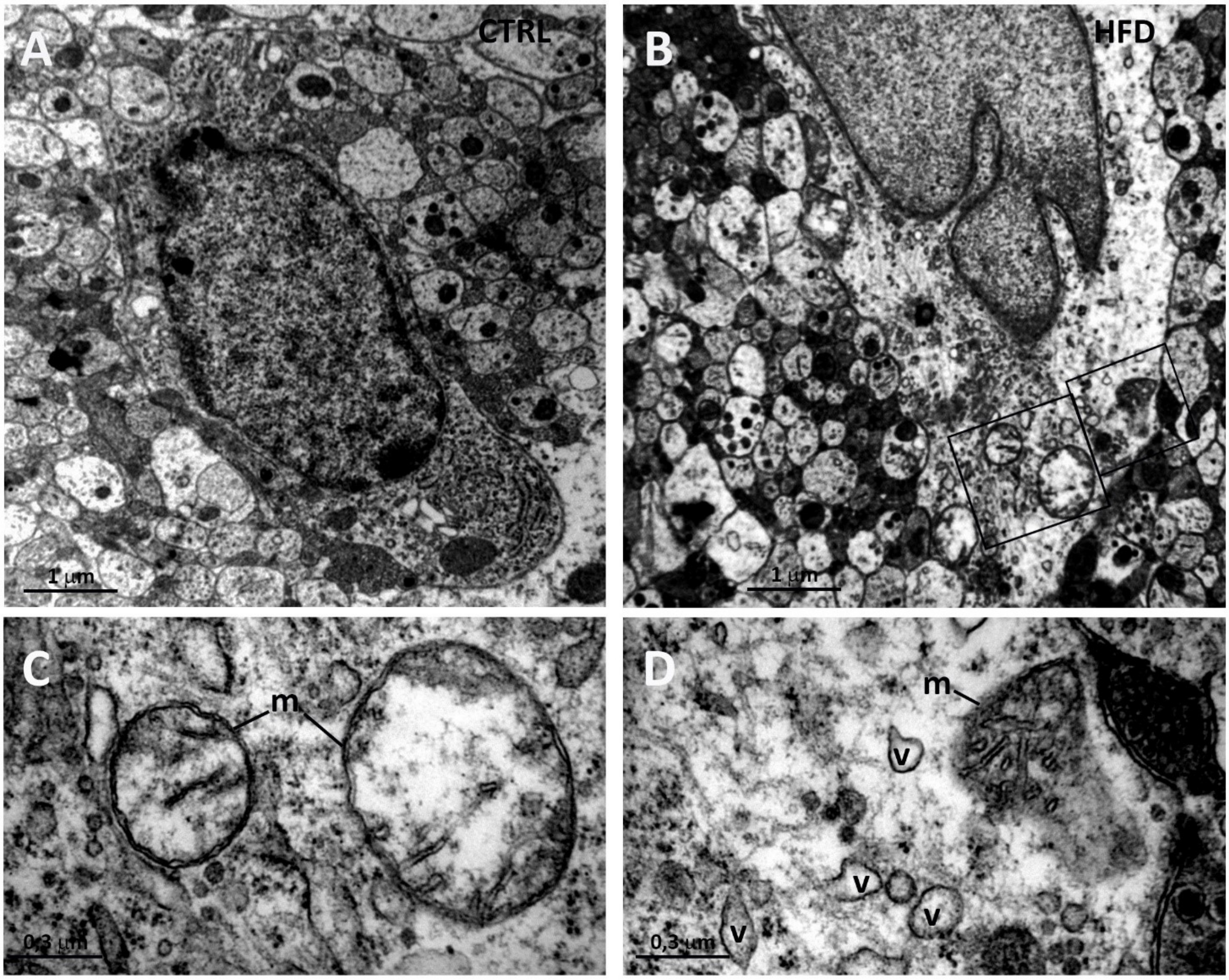

Supplementary Fig. 2: **A** and **B**: transmission electron microscopy of the median eminence subependymal space from a CTRL mouse (A) and from a HFD mouse (B), showing neuronal or glial cell somata surrounded by axonal and dendritic structures. Whereas the cytoplasm of the cell from the CTRL mouse appears electron dense and rich of organelles, the cytoplasm of the cell from the HFD mouse is pale and contains numerous degenerating mitochondria and vesicles. **C** and **D**: enlargements of the areas framed in B, showing degenerating mitochondria (m) and vesicles (v).
