# Supplementary figures and images for: High-Fat Diet Impairs Mouse Median Eminence: A Study by Transmission and Scanning Electron Microscopy Coupled with Raman Spectroscopy
Source: Int J Mol Sci. 2021 Jul 28;22(15):8049. doi: 10.3390/ijms22158049 (PMC8347199; doi:10.3390/ijms22158049)

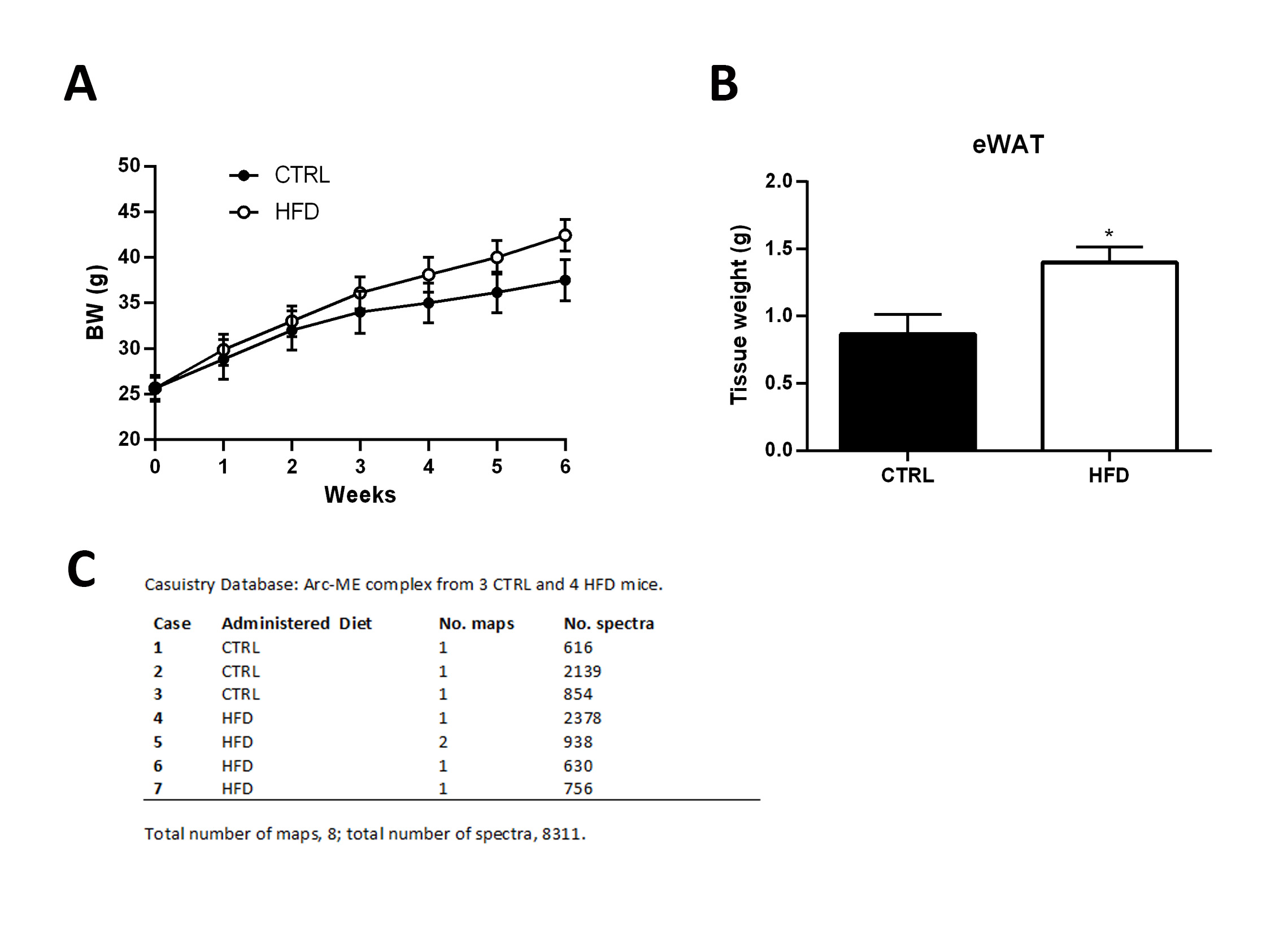

Supplement: Supplementary file 1 [file ijms-22-08049-s001.zip › Suppl. Fig1.jpg]

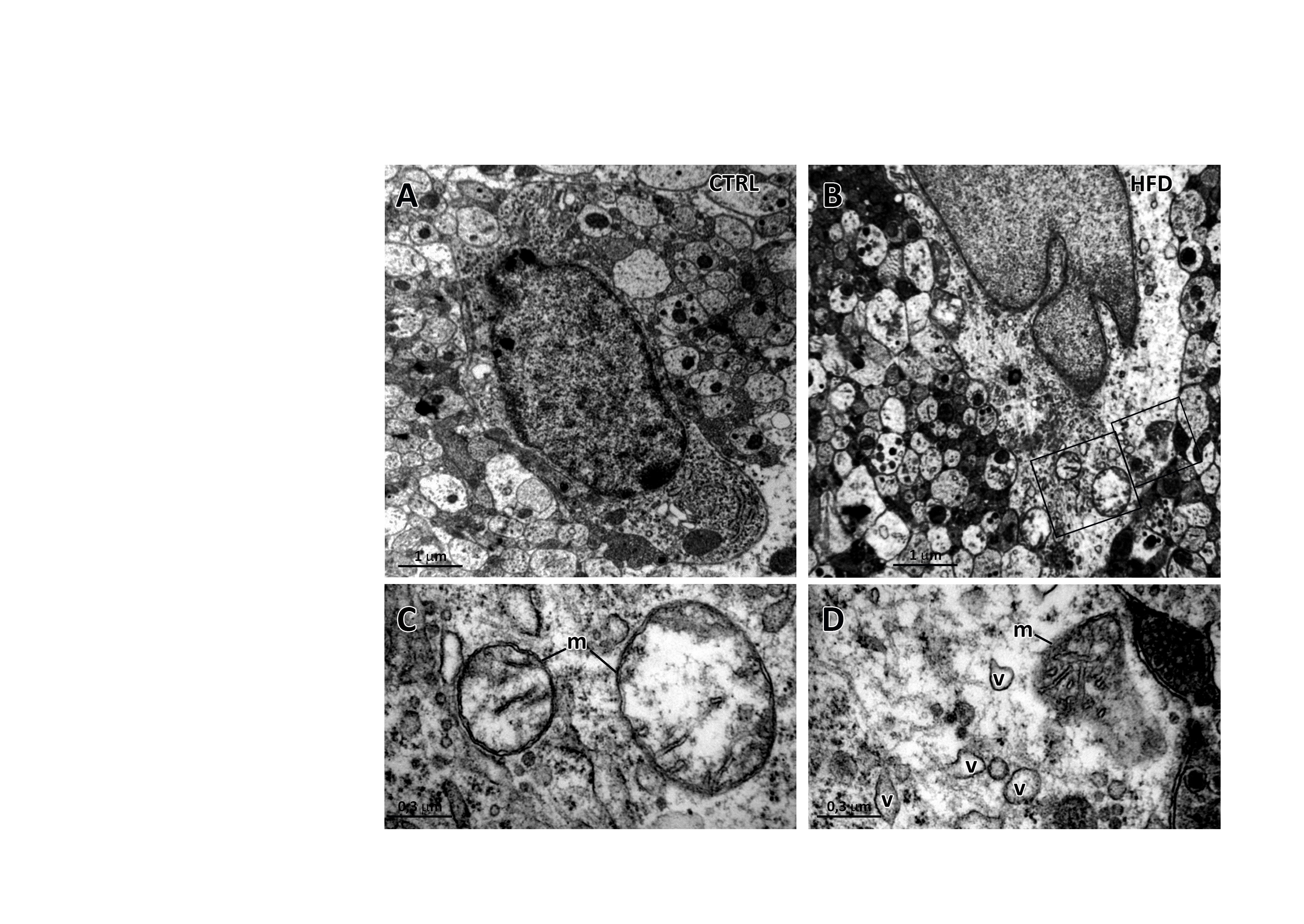

Supplement: Supplementary file 1 [file ijms-22-08049-s001.zip › Suppl. Fig2 revised.jpg]
